# Religion, personality, or none of them? Exploratory evidence on their correlations with economic preference parameters

**DOI:** 10.3389/fpsyg.2024.1361910

**Published:** 2024-08-07

**Authors:** Donata Bessey

**Affiliations:** EastAsia International College, Yonsei University, Mirae Campus, Wonju, Republic of Korea

**Keywords:** big five, risk aversion, time preference, loss aversion, religion, personality

## Abstract

**Introduction:**

Previous empirical research in the social sciences suggests sizable differences across religious denominations for various outcomes of interest, such as educational attainment, marital stability, wealth, or fertility. A small body of previous experimental literature has investigated possible differences in economic preference parameters (including time preference and risk attitude) between religious denominations that might explain those differences.

**Methods:**

This research adds to the extant literature on religion and preferences by including information on subjects’ Big Five personality traits and analyzing potential correlations with loss aversion. It combines experimental data from incentivized choices with information on religious affiliation during high school and Big Five personality traits to test for possible correlations of religious denomination with risk attitude, time preference, and loss aversion, using Bayesian analysis of variance (ANOVA) and Bayesian regression analysis.

**Results:**

Bayesian ANOVA results suggest no preference differences between the religions analyzed in this research. When controlling for Big Five personality traits and a host of other background variables, Bayesian regression results suggest no effects of either religious affiliation or Big Five personality traits measures on the three economic preference parameters analyzed here.

**Discussion:**

These findings highlight the complexity of the relationship between religion, personality traits, and economic preference parameters, suggesting that previously observed differences may be influenced by the preference measures used or other unobserved factors.

## 1 Introduction

This article explores the potential link between religious affiliation and experimentally measured economic preferences, specifically risk attitude, time preference, and loss aversion. Unlike previous research, it also includes information on Big Five personality traits openness, conscientiousness, extraversion, agreeableness, and neuroticism (Goldberg, 1971, 1993), as control variables. This addition allows to account for their potential influence on preferences, contributing to a more comprehensive and nuanced understanding of their interrelationships. Moreover, this study fills a gap in the literature as one of the few to investigate these differences using incentivized experimental choices. If there are indeed differences between followers of religions in economic preference parameters, such as time preference, this finding might add to our understanding of the reasons for differences in outcomes of economic interest, such as educational attainment, beyond the explanations based on social norms and prescriptions regarding education in the different religions discussed below. From a human capital theory perspective (Becker, 1962), lower time preference (i.e., higher levels of patience) predicts higher levels of educational attainment, *ceteris paribus*, because its future payoffs are discounted at lower rates by a rational agent investing in education. Since differences in educational attainment translate into differences in health, lifetime earnings, wealth accumulation, and other important outcomes (Tamborini et al., 2015), research into determinants of its determinants is of key importance to both researchers and policy-makers.

In recent years, economic research has recovered its long-lost interest in culture as a possible determinant of economic outcomes. Early social scientists from Adam Smith to Max Weber were comfortable using cultural differences to explain economic outcomes. The most famous example is Max Weber’s “The Protestant Work Ethic and the Spirit of Capitalism.” However, mainstream economic theory in the 20th century, with its tendency toward representative agents and endogenization, tended to leave little space for cultural factors as possible determinants of economic outcomes. An exception was the work by Karl Polanyi, which was highly influential in sociology and political science but largely ignored by economists. In the mid-nineties, however, economists “discovered” the concept of trust, and subsequent work analyzed the impact of culture on various economic outcomes.

Research results suggest that “culture” (in most economic research loosely defined as ethnicity or religion) is a powerful determinant of economic outcomes. On a macroeconomic level, recent surveys aim to analyze transmission channels and patterns (Iyer, 2016; Basedau et al., 2018). On a microeconomic level, empirical results from the United States suggest considerable differences exist in various outcomes, such as education, fertility, and marriage stability, for different religions. From a theoretical point of view, Becker and Mulligan (1997) analyze the endogenous formation of time preference and discuss the possibility that belief in an afterlife (heaven) might lead individuals to invest more in future-oriented capital, which might lead to less discounting of the future.

Results from prior investigations utilizing datasets from the United States indicate significant differences among various religious denominations. Generally, findings suggest that Jewish Americans boast the highest average level of education. Conversely, fundamentalist Protestants, encompassing exclusivist groups including Baptists, Jehovah’s Witnesses, Seventh-Day Adventists, and Christian Scientists, exhibit the lowest average education levels. Catholics and mainstream Protestants, such as Episcopalians, Methodists, Presbyterians, Lutherans, Unitarians, and other ecumenical bodies, fall within the middle of the educational attainment distribution (Lehrer, 1999). Differences are also reported for other outcomes, such as marital stability, fertility, and women’s labor market participation (Lehrer, 2004).

Several interpretations, grounded in the characteristics of these religions, have been proposed to explain these patterns. For Judaism, these include an argument based on the emergence of social norms around 200 AD (Botticini and Eckstein, 2007), a conjecture that the portability of investments in education, compared to physical investments, appealed to the faith’s members due to their history of persecution (Brenner and Kiefer, 1981), and lower fertility and employment rates among Jewish mothers enhancing the productivity of formal education (Della Pergola, 1980; Chiswick, 1986). The case of Protestantism is intriguing due to the notable divergence in behavior between fundamentalists and mainstream Protestants. Martin Luther himself advocated for universal schooling to enable Christians to read the Bible independently. As of 1970, a higher prevalence of Protestants in European countries correlated with increased gender equality in years of education (Becker and Woessmann, 2008). However, fundamentalist Protestants’ belief in the inerrancy of the Bible and unwillingness to use the scientific method (Darnell and Sherkat, 1997) results in resistance of fundamentalist parents to allow their children’s participation in college-preparatory high school classes that emphasize content perceived as conflicting with “the Biblical truth” (Sherkat and Darnell, 1997). Moreover, fundamentalist colleges are limited and expensive, contributing to a less educated fundamentalist population (Lehrer, 1999). While Lenski’s (1961) work proposed that Catholics generally have lower educational attainment than Protestants, attributing this to the faith’s focus on the afterlife and anti-intellectual orientation, subsequent sociological research has yielded inconsistent results or no support for his theories (Darnell and Sherkat, 1997). Presently, the behavior of Catholics and mainstream Protestants in the United States appears similar concerning fertility, marital stability, and educational attainment (Lehrer, 2004).

Besides social identity and norms, which are likely contributing factors to differences in outcomes of microeconomic interest, religion, and especially religious attendance, change an individual’s preferences, such as risk and time preference. Becker and Mulligan (1997) propose a theoretical model of endogenous determination of time preference (but not risk attitude) where individuals can invest in future-oriented capital (the ability to imagine the future), which will then reduce their discounting of future payoffs. One implication of their analysis is that individuals who expect positive utility after their death, for example, because they believe that they will go to heaven, will invest more in the ability to imagine the future. However, those who believe in negative utility after death (hell) will have fewer investment incentives. Therefore, their model has no precise predictions concerning religious attendance unless believers think that religious attendance alone will increase their odds of going to heaven. Finally, religious attendance might have other effects on individuals’ preferences besides changing individuals’ incentives to invest in future-oriented capital because of their beliefs in a type of afterlife. For example, attending lengthy religious services, praying, meditation, or other religious practices might make an individual more patient or less risk-seeking. These changes in preferences might then translate into different outcomes concerning educational attainment, marital stability, or fertility for followers of different religious denominations and the religiously unaffiliated.

This research tests for such possible correlations of religious affiliation with economic preferences and finds no correlations between religious affiliation and risk attitude, time preference, or loss aversion. In addition, the literature on correlations of Big Five personality traits and various measures of economic preference parameters reveals correlations between Big Five personality traits and risk attitudes, with conscientiousness and openness frequently emerging as significant, providing the rationale to include them in this analysis. However, this research does not replicate any of these previous findings regarding personality traits.

## 2 Literature review

Despite the central importance of both risk attitude and time preference in many economic decision-making models and evidence for considerable heterogeneity of both measures among individuals, only scant research has been carried out on analyzing their determinants in incentivized experiments. One exception is gender, where earlier studies suggested that women are more risk-averse than men (see Croson and Gneezy, 2009 and the references cited in their article), while more recent research suggests that those differences might be task-specific (Filippin and Crosetto, 2016). In addition, a meta-analysis with calculations of effect sizes (Nelson, 2015) shows gender differences that are statistically significant, yet “small” according to Cohen’s criteria (Cohen, 1988), nevertheless providing the rationale to include information on gender in the following statistical analysis.

The following literature survey synthesizes the two strands of literature on correlations of religious affiliation and Big Five personality traits with economic preference parameters. It also included studies that analyzed the effects on risk attitude measured by hypothetical questions, but not experimental research that analyzes the effects of religion in games or the corporate finance literature that analyzes religion’s effects on corporate risk-taking behavior.^1^

The first part summarizes the previous literature on religion and economic preference parameters, beginning with research on risk attitudes. For the United States, Barsky et al. (1997) are an early study to focus on ethnicity and religion as determinants of risk attitude and use answers to hypothetical questions by respondents of the Health and Retirement Study. They find that men are more risk-tolerant than women, Whites in their sample are less risk-tolerant than all other ethnicities, Protestants are least risk-tolerant, and Jewish respondents are most risk-tolerant, with Catholics at the center of the distribution. In contrast, Halek and Eisenhauer (2001) use data on life insurance purchases from the same data set and find no effects of being Catholic, Jewish, or Protestant on individuals’ risk attitudes. Benjamin et al. (2016) employed priming techniques to investigate the impact of religion on risk aversion in an incentivized experiment in the US. Their results showed that priming made Catholics less risk-averse compared to the religiously unaffiliated, with no discernible effect for Protestants. For time preference, no significant effects of priming were found.

For various countries, Miller (2000) analyzes the impact of religious affiliation, attending religious services, and finding comfort in religious services on self-assessed risk propensity on a 10-point scale between 1 (it is best to be cautious when making decisions in one’s life) and 10 (acting boldly is best) in five different countries (India, Italy, Japan, Turkey, and the United States), using data from the World Values Survey. He finds effects of religion on this scale in societies where exclusive, monotheistic religions dominate (Christianity and Islam). In contrast, he finds no effects in societies where non-exclusive religions dominate (Buddhism and Hinduism). Chai et al. (2010) used incentivized choice tasks in an experiment with non-student subject pools in the Philippines and reported no differences between Muslim and Christian participants in risk attitude and time preference. Lastly, Ayifah et al. (2022) use data from Ghana and incentivized choices to analyze the relationship between religion and risk attitudes. They find that any religious affiliation (including Catholicism, Islam, Pentecostalism, Protestantism, and traditional religion), as opposed to being religiously unaffiliated, decreases the probability of engaging in a risky bet. On the contrary, Kahsay et al. (2022) use data from Ethiopian farmers and incentivized choices from the Holt and Laury (2002) risk preference elicitation task and find that in both OLS and instrumental variables regressions, where they use the distance to the nearest relevant place of worship as an instrument for religiosity, it actually increases the number of risky choices in the lottery task.

For Germany, Bartke and Schwarze (2008) analyze the impact of both ethnicity and religion on self-assessed risk propensity on a risk scale between 0 (risk-averse) and 10 (fully prepared to take risks) that has no reference to any specific risk dimension using the German Socio-Economic Panel. They find that Muslims and Catholics score significantly lower on this scale. In contrast, the religiously unaffiliated score significantly higher than Protestants, and they find no impact of ethnicity when controlling for a host of background variables such as education and marital status. Similarly, Dohmen et al. (2011) use data from the German Socio-Economic Panel (SOEP) and respondents’ answers to a general risk-taking question and several domain-specific questions related to the willingness to take risks. They find that compared to Protestants, being Catholic or being affiliated with other Christian as well as non-Christian religions is correlated with lower scores on the general risk-taking question, while being religiously unaffiliated is correlated with higher scores. Lastly, León and Pfeifer (2017) use data from the German Socio-Economic Panel (SOEP) and respondents’ answers to a general risk-taking question, a financial risk-taking question, and information on the types of assets they hold and find that being affiliated with Catholicism, Islam, Protestantism, and other Christian religions, compared to the religiously unaffiliated, significantly negatively correlates with individual general risk-taking attitudes. However, when analyzing the correlations for the financial risk attitude question, they find that being Catholic or Protestant is positively correlated with the willingness to take risks. In addition, when analyzing the correlations for different types of financial assets, they report that Catholics and Protestants are actually more likely than the religiously non-affiliated to hold securities of listed firms and assets of non-listed firms. At the same time, Muslims are less likely to do so.

For the Netherlands, Renneboog and Spaenjers (2011) use survey data and hypothetical questions on individual attitudes toward risk, time preference, and other variables. They find that compared to the religiously unaffiliated, both Catholics and Protestants have longer planning horizons. In addition, they also find that Catholics are more risk averse. In sharp contrast, Noussair et al. (2013) examined the correlation between religion and risk attitudes using a representative survey sample of the Dutch population. Their findings revealed that individuals with stronger religious affiliations, measured by church membership or attendance, tended to be more risk-averse regarding monetary risks, and Protestants displayed higher risk aversion than Catholics.

Regarding time preference, Renneboog and Spaenjers (2011) find that compared to the religiously unaffiliated, both Catholics and Protestants have longer planning horizons. Paglieri et al. (2013) conducted a study utilizing hypothetical intertemporal choice tasks to compare individuals’ decision-making from different religious backgrounds in Italy and the Netherlands. The results indicated variations in patience levels, with Dutch Calvinists demonstrating greater patience than Italian Catholics and Dutch atheists. Italian Catholics exhibited less patience than Dutch Catholics or Italian atheists, while no significant differences were observed among atheists from both countries.

Given the mixed evidence presented for possible correlations between religious affiliation and economic preference parameters presented above, including contradictory findings for hypothetical questions in the same country (such as in the case of the Netherlands), it is almost impossible to provide a summary. Omitted variable bias might explain the mixed evidence, with personality structure being one candidate omitted variable. Hence, the subsequent section provides a concise overview of the literature exploring the interconnections between personality traits and risk measures. Studies using both incentivized and non-incentivized choices reveal various correlations between Big Five personality traits and risk attitudes, with conscientiousness and openness frequently emerging as significant.

Among the studies using non-incentivized choices, Aren and Hamamci (2020) analyzed the effects of financial literacy, Big Five personality traits, and emotions on risk aversion, risky investment intention, and investment choices using a hypothetical choice scale with a convenience sample of the Turkish population. Their risk aversion measure was defined as subjects’ answers to a 7-item risk aversion, and they found that neuroticism and openness are correlated with this scale. Brooks and Williams (2021) utilized the ‘ATR-15′ questionnaire to measure risk attitude and studied correlations with Big Five personality traits. They reported a negative correlation between Big Five Neuroticism and their risk measure in ordered probit regressions containing control variables for emotions, gender, education, occupational status, marital status, income, and wealth. Similarly, Aumeboonsuke and Caplanova (2021) explored correlations between Big Five personality traits and the TSI (Thai Securities Institute) Risk Profile Questionnaire consisting of 10 multiple-choice questions, concluding that conscientiousness and openness positively correlate with risk aversion. The following section summarizes the research using incentivized choice tasks in experiments or measures derived from actual investment behavior as risk measures. Becker et al. (2012) used three samples, including incentivized choice tasks, to analyze correlations with Big Five personality traits. They found different coefficient signs and strengths of correlations between the three data sets, suggesting that personality traits and economic preferences cannot substitute for each other. Prinz et al. (2014) used experimental data on incentivized risk elicitation tasks from a student sample to analyze the relationship between Big Five personality traits and reported no statistically significant correlations. Rustichini et al. (2016) used experimental data from a sample of truck driver trainees and information on Big Five personality traits derived from the Multidimensional Personality Questionnaire (MPQ) and incentivized choices on a risk attitude and time preference elicitation task. They found that neuroticism positively correlates with risk aversion, with no significant correlations for time preference. Bucciol and Zarri (2017) used data from the 2006–2012 United States Health and Retirement Study waves. They analyzed the personality-related correlates of financial risk-taking, as measured by the probability of holding any stock assets and their amount, and reported that Big Five agreeableness negatively correlates with the probability of holding stocks, which they interpret as evidence of lower risk aversion. Engle-Warnick et al. (2020) used experimental data and incentivized risk (and ambiguity) tasks and analyzed participants’ chat contents to derive information on their personality traits in the Big Five model. They found a positive correlation between their measure of conscientiousness and risk aversion. Piovesan and Willadsen (2021) used experimental data from a gift-incentivized experiment with children. They found no correlations between personality traits, as measured by the HEXACO questionnaire, and their risk measure when using one personality trait at a time as a predictor in a series of regressions. Firth et al. (2023) used responses to a self-report survey. They matched administrative data from a brokerage firm to investigate the relationships between Big Five traits and IQ and individuals’ stock trading portfolios. They reported that Big Five Conscientiousness has positive associations with overconfidence and investment performance, while openness has positive associations with overconfidence and idiosyncratic risk, and extraversion has positive associations with overconfidence and negative associations with idiosyncratic risk and investment performance.

As this review of the scarce existing literature on religion and economic preference parameters has shown, the empirical and experimental evidence for the effects of specific religious affiliations on risk preference and time attitude is mixed, even when identical tasks are being used for elicitation. Possible reasons include the use of conceptionally diverse measures of preference parameters, the likely effects of culture beyond religion in cross-country comparisons, the use of different samples of both student and non-student populations, and possible omitted variables bias in model specifications. In addition, the research using incentivized economic experiments is scant. The rationale for their preferability in experimental economic research lies in the possibility that choices in incentivized vs. hypothetical tasks might differ (Anderson and Mellor, 2008) and that self-reported attitudes might differ from actual choices (Glaeser et al., 2000). Finally, and probably the key reason for their use, experimental tasks provide standardized measures of key preferences that facilitate benchmarking and replication, and financial incentives may alleviate response biases (Hoffmann, 2013).

To the best of the author‘s knowledge, no previous research has included information on religious affiliation and personality traits as determinants of economic preference parameters simultaneously. Since the previous evidence on either possible set of determinants is far from conclusive, this research adopts an explorative approach and refrains from positing testable hypotheses.

## 3 Materials and methods

Data were collected in an online experiment. Subjects were undergraduate students in two-and four-year California colleges recruited online and on campuses. In total, the data set contains information on *n* = 110 subjects.

First, subjects gave their written informed consent and answered a questionnaire containing items on their socio-economic background, for example, parental education, hobbies, friends and family structure, and, most notably for this research, religious affiliation. Subsequently, the survey included a 15-item version of the Big Five inventory. The second part of the experiment consisted of incentivized choice questions to elicit subjects’ risk attitudes and time preferences. In the last part, payments were determined, and subjects provided their postal addresses to receive the payment for their participation.

This research used incentivized choices to elicit subjects’ risk and time preferences. Subjects received a show-up fee of USD 10 because the choice questions also included a question to elicit loss aversion. The questionnaire and the entire script and instructions used in the experiment are provided in the appendix. Subjects were paid by checks sent by certified mail to the postal address they provided. On average, the experiment and survey duration was 47 min, and the average payment was US$39. Since this was substantially above the relevant hourly minimum wages (both for the state of California and city and county-level minimum wages), the payment should have provided sufficient incentives for participation.

Since the experiment and questionnaire were designed to contain information on preference parameters, religious affiliation, and personality traits, the obtained data are uniquely suited to analyze the research question.

### 3.1 Lotteries, discounting choices, and loss aversion elicitation

In the online experiment, all subjects had to read instructions for the choice questions, including information on how they would be paid and how the relevant payment decisions would be determined. For the risk preference elicitation tasks, participants were informed that one row of their choices would be randomly selected to be relevant for their payment. For the time preference elicitation tasks, one out of 10 participants would be paid according to their choices, and subjects were informed at the last stage of the experiment if they were chosen to be paid. As in the risk preference elicitation task, the relevant payment was determined by randomly selecting one row to be relevant for payment, but only one-tenth of the subjects were paid. These randomization procedures incentivize subjects to choose according to their preferences in each choice task, and paying them after the experiment avoids endowment effects. The instructions also contained the information that subjects would receive all payments from the risk questions and the intertemporal choice questions as a check by certified mail at the relevant payout dates chosen (immediately for risk elicitation tasks and if they preferred the immediate payment in the intertemporal choice task, or at the relevant point in the future, had they chosen the deferred payment).

This research elicited subjects’ risk attitudes using choices between a paid lottery and safe payments. Participants made choices in a sheet with 10 rows, deciding between a safe option (the certainty equivalent) or playing the lottery in each row. They could win USD 10 or USD 0 in the lottery, with a probability of 50% each. The lottery did not change for the different rows, but the amount participants would receive if they chose the safe option would increase from row to row (USD 1 to USD 10). Upper and lower bounds for individuals’ degree of risk aversion can then be calculated assuming a functional form for their utility function (such as constant relative risk aversion, CRRA) and using the safe options from the switching row and the previous row in the experiment. The individual degree of risk aversion is then given by γ = 1 - ln(p)/[ln(y) - ln(x)], where p denotes the winning probability in the choice lottery, y denotes the safe option, and x denotes the winning payment from the lottery. However, for the sake of simplicity, this research used the number of safe choices an individual made as a measure of their risk attitude in regressions.

For measuring subjects’ time preference rates, this research used one more choice sheet with 20 rows for choices between receiving payments at different times. Again, subjects were asked to make choices in each row of a decision sheet. In the intertemporal choice question, the choice was between USD 100 in 3 months and a smaller amount X that subjects would be paid immediately. The size of the immediate payment X increased by USD 5 in each row, starting from a value of USD 5 in the first row up to a value of USD 100 in the 20th row. Bounds of individuals’ degree of time preference can then be calculated using the immediate payments from the switching row and the previous row. Bounds for the individual’s degree of time preference r are then be given by 1 + r = ∛(y/x)1 + r where r denotes time preference, y denotes the delayed payment, and x denotes the immediate payment. However, for the sake of simplicity, this research used the number of patient choices an individual made as a measure of their time preference in regressions.

Regarding loss aversion, a measure developed by Fehr and Goette (2007) in the adaptation of Gächter et al. (2007) was used. In this choice task, subjects decided whether to accept or not six lotteries over gains and losses. In each lottery, the gain outcome was identical (US$6) and the loss outcome increased between $2 and $7 with a 50% probability of each outcome. If subjects rejected a lottery, their payment was zero. At the end of the experiment, only one lottery was selected randomly for pay (Cubitt et al., 1998). The decision sheet can be found in Appendix B. As a measure of loss aversion in regressions, the number of lotteries the subject declined was used, with higher values corresponding to higher degrees of loss aversion.

### 3.2 Big Five inventory 15-item short version

The first part of the study also included the background questions and a 15-item short version of the Big Five inventory.

The Big Five are five dimensions that define human personality at the broadest level (Goldberg, 1971; Goldberg, 1993). For measurement of the personality dimensions (Openness to experience, Conscientiousness, Extraversion, Agreeableness, and Emotional Stability), this research used a 15-item short version of the Big Five inventory developed and validated for inclusion in the German Socio-Economic Panel (Gerlitz and Schupp, 2005; Lang et al., 2005). It was aggregated identically to the original research but not standardized. See the appendix for the items used.

### 3.3 Socio-economic background questionnaire

The background questionnaire contained the following questions included in regression analysis: a question on self-reported gender, as previous evidence suggests that there might be differences in risk-taking behavior between men and women (Croson and Gneezy, 2009). Information on self-reported ethnicity was used to create a dummy variable that takes the value of 1 if someone is African/Asian American, Hispanic/Latino, or Native/Hawaiian as opposed to Caucasians as the reference group. Also, a dummy variable that takes the value of 1 if someone is a high school graduate instead of a GED holder was used as a rough measure of cognitive ability following (Falk et al., 2010). As measures of family background, the following information was included: mother’s and father’s education in seven categories (“other” or “unknown education,” some high school, high school, some college, associate’s degree, bachelor’s degree, graduate degree), a dummy variable that takes the value of 1 if a respondent has at least one sibling as opposed to being an only child and, as a very rough income measure, the respondent’s answer to the following question: “On a scale from 1 to 5, how difficult is it for you to acquire USD 100 for personal spending?,” with 1 = “very hard” and 5 = “not hard at all.”

### 3.4 Statistical methods

Bayesian ANOVA was used to investigate possible differences in risk preference, time attitude, and loss aversion between religious affiliations. This choice was motivated by several factors. Firstly, Bayesian analysis provides a flexible framework for model comparison, allowing to assess the relative support for competing hypotheses using Bayes factors. Unlike traditional frequentist approaches, which rely on *p*-values and null hypothesis significance testing, Bayesian methods offer a more intuitive way to compare models and quantify evidence for or against different hypotheses. In addition, Bayesian ANOVA offers a straightforward approach to model comparison, which is particularly advantageous when comparing multiple groups. Additionally, Bayesian analysis allows for incorporating uncertainty in parameter estimates, providing a more complete picture of the data and avoiding overconfident interpretations. This is especially important when dealing with small sample sizes or heterogeneous populations, where traditional frequentist methods may yield unreliable results. Overall, it offers a powerful and flexible framework for investigating group differences while accounting for uncertainty and providing intuitive measures of evidence for competing hypotheses.

Appendix Tables A3, A4 provide the findings from classical ANOVA and non-parametric classical hypothesis tests (specifically, Kruskal-Wallis tests), confirming the Bayesian ANOVA analysis results.

The classification of Bayes factors was conducted according to the framework established by Andraszewicz et al. (2015), which is derived from Jeffreys’s (1961) original work and replaced some of the original labels. This classification aims to facilitate scientific communication based on categorizing Bayes factors according to their size. In this framework, a Bayes factor BF_01_ suggests “extreme” evidence for M_0_ (i.e., the null model) if its size is above 100 (and, vice versa, extreme evidence for M_1_, the alternative model, if its size is below 1/100), “very strong” evidence for M_0_ if it is between 30 and 100 (and, vice versa, very strong evidence for M_1_ if its size is between 1/100 and 1/30), “strong” evidence for M_0_ if it is between 10 and 30 (and, vice versa, strong evidence for M_1_ if its size is between 1/30 and 1/10), “moderate” evidence for M_0_ if it is between 3 and 10 (and, vice versa, moderate evidence for M_1_ if its size is between 1/10 and 1/3), and “anecdotal” evidence for M_0_ if it is between 1 and 3 (and, vice versa, anecdotal evidence for M_1_ if its size is between 1/3 and 1). While this categorization facilitates communication, readers should remember that they are only “an approximate descriptive articulation of different standards of evidence” (Andraszewicz et al., 2015).

Bayesian ANOVA was performed using JASP (JASP Team, 2023), an open-source statistical software program based on R. It adheres closely to the guidelines outlined by Rouder et al. (2012) for specifying and implementing default Bayes factor tests that are computationally convenient as well as invariant to measurement units, while also facilitating result interpretation (van den Bergh et al., 2020). Given the exploratory nature of the research question under examination, no alterations were made to JASP’s default prior distributions, per the recommendations provided by van Doorn et al. (2021). JASP yields Bayes factors for both null and alternative models, and the shifts in predictive power from the null to the alternative model are discussed in the subsequent analyses.

In a first step, the Bayesian ANOVA compares the null model (no effect of religion) with the alternative models (effect of religion, all groups combined). This comparison is based on the Bayes factors outlined above. If this Bayes factor indicates evidence for the alternative model over the null model, post-hoc tests are performed to identify specific differences between individual religious groups and the non-affiliated group. This approach allows for a comprehensive understanding of the potential differences between different religions in economic preference parameters.

In addition, regression results from Bayesian regressions for the determinants of risk preferences are presented to investigate additional possible effects. These regressions were run in STATA 17.0 using the bayesmh command. For methodological consistency, the same uninformative priors as in JASP [Cauchy (0, r = 1/√2)] were implemented in Stata [which by default uses normal priors (0, 10,000) for the regression coefficients and an inverse-gamma prior with shape and scale parameters of 0.01 for the error variance]. This prior is a common default choice in applications because it strikes a balance between being weakly informative and providing regularization. The dependent variables (risk attitude, time preference, and loss aversion) were regressed on a set of independent variables (measuring religious affiliation, gender, ethnicity, previous schooling, maternal and parental education, an income measure, if the respondent has any siblings, and the Big Five personality traits).

Bayesian estimation using Markov Chain Monte Carlo (MCMC) methods was used. It estimates the posterior distribution of the coefficients given the prior distributions and the data. To generate initial values for multiple chains in Stata and improve their convergence, the following strategy was used to obtain slightly different initial values for all 16 chains: first, an OLS regression was performed, and the parameter estimates were then slightly disturbed for each chain. This strategy of adding a small random perturbation for multiple chains in Bayesian analysis is commonly recommended to ensure good mixing and convergence of the chains, for example by Gelman et al. (2014). The idea is that starting from different initial values can help diagnose whether the chains are mixing well and converging to the same posterior distribution. Each chain was sampled to explore the posterior distribution of the model parameters. The likelihood function was assumed to be normal for the dependent variables, given the observed data and the estimated coefficients. The MCMC sample size was set to 1,000,000, and the total number of iterations for the MH algorithm equals the sum of the burn-in iterations (10,000), which are discarded, and the MCMC sample. This sample size applies to each of the 16 chains.

### 3.5 Operationalization of variables and specification of regression models

The dependent variables were measured as follows. To elicit risk attitude, respondents chose between a lottery with a 50% chance of paying US$ 10 and safe payments increasing from US$ 1 to US$ 10. The number of safe choices served as a measure of risk attitude. To elicit time preference, subjects chose between US$ 100, paid three months after participating in the experiment, and immediate payments increasing from US$ 5 to US$ 100. The number of deferred choices, i.e., choices of the payment of US$ 100 three months after the experiment instead of the immediate payment, served as a measure of time preference.

Religious affiliation was measured as respondents’ answers to a question about their religious affiliation with the following answer options: Buddhism, Catholicism, Hinduism, Judaism, Islam, Sikh, Mormon, Protestantism (including various Protestant denominations), and Other (with a free-form answer option). Those who stated no religious affiliation were used as the reference group. Due to the small number of respondents, one Hindu and one Mormon observation were excluded from the ANOVA analysis.

Beyond those regressors of interest, namely, a respondent’s religious affiliation and the intensity with which they attended religious events during their high school years, the following control variables for demographic characteristics were used: an ethnicity measure, as discussed above, and a dummy variable that takes the value of 1 if the respondent is a high school graduate as opposed to holding a GED as an imperfect measure of cognitive ability.

To control for respondents’ family background, the following control variables were added: mother’s and father’s education (“other” or “unknown education,” some high school, high school, some college, associate’s degree, bachelor’s degree, graduate degree on a scale from 0 to 6), an income measure as the answer to the following question: How hard is it for you to acquire US$ 100 for personal spending? on a scale from 1 to 5 (with 1 = very hard and 5 = not hard at all) and a dummy variable that takes the value of 1 if someone has any siblings as opposed to being an only child.

Finally, all regressions contained information on respondents’ Big Five personality traits (openness, conscientiousness, extraversion, agreeableness, and neuroticism).

## 4 Results

The following section discusses descriptive statistics for the experimental sample and results from Bayesian analysis of variance and linear regression.

Complete descriptive statistics and a correlation matrix for all variables are reported in the appendix.

Table 1 shows descriptive statistics for the sample.

**Table 1:** Descriptive statistics.

Variable	Mean	Standard error of the mean	Min	Max
1 = Buddhism	0.0636	0.0234	0	1
1 = Catholicism	0.2364	0.0407	0	1
1 = Hinduism	0.0091	0.0091	0	1
1 = Islam	0.0364	0.0179	0	1
1 = Judaism	0.0273	0.0156	0	1
1 = Mormonism	0.0091	0.0091	0	1
1 = Protestantism	0.1727	0.0362	0	1
1 = female	0.4455	0.0476	0	1
1 = non-white	0.5273	0.0478	0	1
1 = high school graduate	0.8727	0.0319	0	1
Income measure	3.3909	0.1355	0	5
1 = any siblings	0.8455	0.0346	0	1
Big Five openness	5.7909	0.1156	1	7
Big Five conscientiousness	5.4879	0.0979	3	7
Big Five extraversion	4.7879	0.1223	1.6667	7
Big Five agreeableness	5.6515	0.1156	1	7
Big Five neuroticism	3.6818	0.1405	1	7

The largest religious group in the sample is Catholics, with almost 24%, followed by various Protestant denominations, with around 17%. Next come Buddhists and Muslims with over 6%, or 4 respondents each, and Jews with almost 3% (2 respondents). The largest group overall is the religiously unaffiliated, with about 47.27%.

Regarding socio-economic characteristics, about 45% of the sample are female, and a slight majority of almost 53% report a non-white ethnicity. More than 87% are high school graduates as opposed to being „Certificate of High School Equivalency” (GED) holders. The mean answer to the income measurement question was 3.39, with 5 being the mode category (chosen by 32 respondents). Lastly, almost 85% of respondents report having at least one sibling.

Table 2 shows descriptive differences in time preference, risk attitude, and loss aversion between religious groups and the religiously unaffiliated. It shows differences between the groups to be investigated in the following paragraphs using Bayesian ANOVA and linear (OLS) regression.

**Table 2:** Preference parameters by religion.

	Unaffiliated	Buddhism	Catholicism	Hinduism	Islam	Judaism	Mormonism	Protestantism	Entire sample
Risk attitude	5.27	4.40	5.88	7	8.00	3.00	4	5.74	5.52
Time preference	12.79	13.40	12.00	17	15.50	14.50	19	10.89	12.53
Loss aversion	3.40	4.00	3.15	4	4.50	4.50	5	3.42	3.45
*n*	52	5	26	1	4	2	1	19	110

The following discussion of the results provided in Tables 3–5 is based on the framework for analysis suggested by Andraszewicz et al. (2015) described in more detail in section 3.4. Please note that the Hindu and the Mormon observations could not be used in this part of the analysis since there was just one observation each. Tables 3–5 the following abbreviations are used: P(M) = prior model probability. P(M|data) = posterior model probability, i.e., updated probability of model, given the data. BF_M_ = posterior model odds = $\frac{P\left(M|D\right)}{1-P\left(M|D\right)}\times \frac{1-P\left(M\right)}{P\left(M\right)}$ , or the change from the prior odds to the posterior odds for the model. BF_01_ = The Bayes factor BF_01_ quantifies evidence for the null hypothesis relative to the alternative hypothesis. This is equal to 1/BF_10_. Error %: The error of the Gaussian quadrature integration routine used by the BayesFactor package, an estimate of the numerical error in the computation of the Bayes factor. According to Van den Bergh et al. (2020), error percentages below 20% are deemed acceptable in many situations.

**Table 3:** Bayesian ANOVA – risk attitude.

Model comparison: risk attitude					
Models	P(M)	P(M|data)	BF_M_	BF_01_	error %
Null model	0.500	0.790	3.766	1.000	
Religions	0.500	0.210	0.266	3.766	3.873 × 10^−4^

						95% credible interval for mean	
		Prior odds	Posterior odds	BF_01, U_	Error %	Lower	Upper
Religiously unaffiliated						4.636	5.903
	Buddhism	3.847	7.433	1.932	9.075 × 10^−4^	2.517	6.283
	Judaism	3.847	4.349	1.13	0.002	−22.412	28.412
	Islam	3.847	1.537	0.4	0.002	4.102	11.898
	Catholicism	3.847	9.507	2.471	0.012	4.842	6.927
	Protestantism	3.847	11.369	2.955	0.009	4.463	7.01
Buddhism	Judaism	3.847	5.465	1.42	0.002		
	Islam	3.847	1.489	0.387	4.446 × 10^−4^		
	Catholicism	3.847	5.506	1.431	9.974 × 10^−4^		
	Protestantism	3.847	6.134	1.594	0.001		
Judaism	Islam	3.847	2.607	0.678	0.007		
	Catholicism	3.847	3.891	1.011	0.004		
	Protestantism	3.847	4.301	1.118	0.004		
Islam	Catholicism	3.847	4.087	1.062	8.470 × 10^−4^		
	Protestantism	3.847	3.93	1.021	0.001		
Catholicism	Protestantism	3.847	12.74	3.311	0.006		

The posterior odds have been corrected for multiple testing by fixing to 0.5 the prior probability that the null hypothesis holds across all comparisons (Westfall et al., 1997). Individual comparisons are based on the default t-test with a Cauchy [0, r = 1/sqrt(2)]a prior. The “U” in the Bayes factor denotes that it is uncorrected. *Post hoc* comparisons: risk attitude.

**Table 4:** Bayesian analysis of variance: time preference.

Model comparison: time preference					
Models	P(M)	P(M|data)	BF_M_	BF_01_	error %
Null model	0.500	0.893	8.378	1.000	
Religions	0.500	0.107	0.119	8.378	0.001

						95% credible interval for mean	
		Prior odds	Posterior odds	BF_01, U_	Error %	Lower	Upper
Religiously unaffiliated						11.294	14.28
	Buddhism	3.847	9.211	2.394	0.001	5.23	21.57
	Judaism	3.847	6.921	1.799	2.372 × 10^−4^	−55.384	84.38
	Islam	3.847	6.442	1.675	5.488 × 10^−4^	8.564	22.44
	Catholicism	3.847	13.385	3.479	0.013	9.662	14.34
	Protestantism	3.847	7.254	1.885	0.008	7.999	13.79
Buddhism	Judaism	3.847	6.693	1.74	1.654 × 10^−4^		
	Islam	3.847	6.909	1.796	6.030 × 10^−4^		
	Catholicism	3.847	8.444	2.195	9.034 × 10^−4^		
	Protestantism	3.847	7.199	1.871	0.001		
Judaism	Islam	3.847	6.569	1.708	1.672 × 10^−4^		
	Catholicism	3.847	6.596	1.714	3.084 × 10^−4^		
	Protestantism	3.847	6.059	1.575	4.761 × 10^−4^		
Islam	Catholicism	3.847	5.642	1.467	7.129 × 10^−4^		
	Protestantism	3.847	4.427	1.151	0.001		
Catholicism	Protestantism	3.847	11.053	2.873	0.006		

The posterior odds have been corrected for multiple testing by fixing to 0.5 the prior probability that the null hypothesis holds across all comparisons (Westfall et al., 1997). Individual comparisons are based on the default t-test with a Cauchy [0, r = 1/sqrt(2)] prior. The “U” in the Bayes factor denotes that it is uncorrected.

**Table 5:** Bayesian analysis of variance: loss aversion.

Model comparison					
Models	P (M)	P (M|data)	BF_M_	BF_01_	Error %
Null model	0.500	0.913	10.459	1.000	
Religions	0.500	0.087	0.096	10.459	0.002

						95% credible interval for mean	
		Prior odds	Posterior odds	BF_01, U_	Error %	Lower	Upper
Religiously unaffiliated						2.863	3.945
	Buddhism	3.847	8.047	2.091	9.514 × 10^−4^	3.122	4.878
	Judaism	3.847	6.165	1.602	3.058 × 10^−4^	−1.853	10.853
	Islam	3.847	5.934	1.542	0.012	2.446	6.554
	Catholicism	3.847	13.647	3.547	0.013	2.462	3.846
	Protestantism	3.847	14.212	3.694	0.009	2.34	4.503
Buddhism	Judaism	3.847	5.6	1.456	0.002		
	Islam	3.847	6.406	1.665	9.706 × 10^−4^		
	Catholicism	3.847	6.22	1.617	9.973 × 10^−4^		
	Protestantism	3.847	8.077	2.099	8.827 × 10^−4^		
Judaism	Islam	3.847	6.655	1.73	1.201 × 10^−4^		
	Catholicism	3.847	5.256	1.366	3.316 × 10^−4^		
	Protestantism	3.847	6.373	1.656	3.163 × 10^−4^		
Islam	Catholicism	3.847	4.228	1.099	7.812 × 10^−4^		
	Protestantism	3.847	6.493	1.688	8.116 × 10^−4^		
Catholicism	Protestantism	3.847	11.894	3.092	0.006		

Bayesian analysis of variance of the null hypothesis that there are no differences in risk preferences between the different religions showed “moderate” evidence for the null hypothesis (BF_01_ = 3.766), suggesting no differences in risk attitude between religions. A post-hoc analysis reveals that when comparing all religions, the Bayes factor BF_01_ in favor of the null hypothesis is 0.400 for the comparison between the religiously unaffiliated and Muslims, indicating “anecdotal” evidence in favor of the alternative hypothesis that there is indeed a difference between them. Bayes factors BF_01_ for the comparisons between all other religions and the religiously unaffiliated range between 1.130 and 2.955, suggesting “anecdotal” evidence favoring the null hypothesis relative to the alternative hypothesis.

Table 4 presents Bayesian analysis of variance for time preference.

Regarding time preference, Bayesian analysis of variance of the null hypothesis that there are no differences between the religions showed „moderate “evidence in favor of the null hypothesis (BF_01_ = 8.378), suggesting no differences in time preference between religions. Consequently, a post-hoc analysis comparing all religions resulted in Bayes factors BF_01_ ranging between 1.675 and 3.499, suggesting „anecdotal“to „moderate“evidence favoring the null hypothesis.

Finally, Table 5 presents Bayesian analysis of variance results for loss aversion.

For loss aversion, Bayesian analysis of variance of the null hypothesis that there are no differences between the religions showed „strong “evidence in favor of the null hypothesis (BF_01_ = 10.459), suggesting no differences in loss aversion between religions. Again, a post-hoc analysis comparing all religions resulted in Bayes factors BF_01_ ranging between 1.542 and 3.694, suggesting „anecdotal“to „moderate“evidence favoring the null hypothesis.

Appendix Tables A.3, A.4 present results from classical ANOVA and Kruskal-Wallis tests, also concluding that no statistically significant differences exist between the religious affiliations analyzed in this research.

Tables 6–8 presents estimation results from Bayesian linear regressions on the relationship between religious affiliation and personality traits with risk attitude, time preference, and loss aversion.

**Table 6:** Bayesian regression: risk aversion.

		95% Credible interval			
	Posterior mean	Lower	Upper	ESS	$\hat{R}$
1 = Buddhism	−0.4106	−8.7540	6.6867	32,567	1.2
(As opposed to relig.unaffiliated)					
1 = Catholicism	0.5558	−6.4204	15.5319	33,055	1.3
(As opposed to relig.unaffiliated)					
1 = Hinduism	0.1466	−7.7052	7.5315	27,755	1.2
(As opposed to relig.unaffiliated)					
1 = Islam	−0.2475	−8.3497	4.6825	31,636	1.1
(As opposed to relig.unaffiliated)					
1 = Judaism	−1.1652	−9.1117	4.5230	32,303	1.1
(As opposed to relig.unaffiliated)					
1 = Mormon	−0.4228	−8.1455	7.6858	28,742	1.1
(As opposed to relig.unaffiliated)					
1 = Protestantism	0.0803	−5.5933	6.3845	27,527	1.2
(As opposed to relig.unaffiliated)					
1 = female	0.0760	−5.9274	5.7099	37,888	1.1
(As opposed to male)					
1 = Ethnicity other than white	0.1080	−4.7098	6.0573	30,926	1.1
1 = High school graduate	0.2516	−7.3625	7.1414	27,780	1.1
(As opposed to GED holder)					
Mothers’ education	−0.0254	−5.6553	5.3716	36,712	1.1
Fathers’ education	−0.2066	−4.9030	4.2887	36,413	1.0
Income measure	0.0295	−4.9133	5.3232	34,862	1.1
1 = any siblings	−0.0817	−8.6314	6.9655	32,267	1.3
(As opposed to only child)					
Big five openness	−0.3791	−10.4768	5.7037	32,176	1.1
Big five conscientiousness	0.7152	−4.0502	7.8849	33,344	1.3
Big five extraversion	−0.0004	−5.5564	5.0734	29,346	1.1
Big five agreeableness	0.2108	−5.3290	7.2634	28,600	1.1
Big five neuroticism	−0.4886	−8.8867	5.6432	40,813	1.1
Constant	2.0555	−5.5886	9.4738	36,597	1.2
Observations	110				

For all religious affiliation dummy variables, the baseline category are the religiously unaffiliated. Mother’s and father’s education are measured on a scale from 0 to 6, with 0 = less than high school or unknown, 1 = some high school, 2 = high school graduate, 3 = some college, 4 = associate’s degree, 5 = college graduate, 6 = graduate degree. The income measure is the answer to the following question: How hard is it for you to acquire US$ 100 for personal spending?, on a scale from 1 to 5 (with 1 = very hard and 5 = not hard at all). ESS denotes the effective sample size for each parameter. It is an estimate of the number of independent samples from the posterior (Kruschke, 2015). The Gelman-Rubin statistic $\hat{R}$ offers a quantitative approach to assess whether chains converge to the same posterior (Gelman and Hill, 2007). By comparing the variability between chains with the variability within chains, $\hat{R}$ determines convergence. When $\hat{R}$ < 1.1, it indicates that the chains have converged.

**Table 7:** Bayesian regression: time preference.

		95% Credible interval			
	Posterior mean	Lower	Upper	ESS	$\hat{R}$
1 = Buddhism	0.6498	−4.4539	7.3299	26,506	1.1
(As opposed to relig.unaffiliated)					
1 = Catholicism	−0.1942	−6.5339	5.6703	35,272	1.1
(as opposed to relig.unaffiliated)					
1 = Hinduism	0.5160	−8.5237	9.5101	26,949	1.2
(As opposed to relig.unaffiliated)					
1 = Islam	1.6149	−4.7907	14.3903	30,862	1.4
(as opposed to relig.unaffiliated)					
1 = Judaism	1.0942	−5.7401	18.8193	25,995	1.4
(As opposed to relig.unaffiliated)					
1 = Mormon	−0.2468	−10.3604	6.5760	26,825	1.3
(As opposed to relig.unaffiliated)					
1 = Protestantism	−0.2246	−7.2012	7.0349	24,899	1.1
(as opposed to relig.unaffiliated)					
1 = female	0.0220	−4.7696	5.2817	32,146	1.1
(As opposed to male)					
1 = Ethnicity other than white	−0.0325	−7.8835	6.0597	30,647	1.1
1 = High school graduate	−0.4692	−8.2123	6.6637	25,419	1.1
(As opposed to GED holder)					
Mothers’ education	−0.2612	−7.1943	4.6201	33,174	1.1
Fathers’ education	−0.0989	−7.1497	6.4786	34,297	1.1
Income measure	−0.2980	−8.1594	6.0988	29,297	1.2
1 = any siblings	0.5827	−5.0188	7.7067	26,097	1.1
(As opposed to only child)					
Big Five openness	0.1164	−5.9113	6.8715	30,165	1.1
Big Five conscientiousness	−0.0234	−4.8027	5.1591	28,216	1.1
Big Five extraversion	−0.3740	−7.3503	5.6328	34,078	1.1
Big Five agreeableness	−0.2719	−7.7124	6.1686	26,996	1.2
Big Five neuroticism	−0.1369	−6.2897	5.4234	31,093	1.1
Constant	5.8457	−4.3511	20.1114	26,930	1.4
Observations	110				

For all religious affiliation dummy variables, the baseline category are the religiously unaffiliated. Mother’s and father’s education are measured on a scale from 0 to 6, with 0 = less than high school or unknown, 1 = some high school, 2 = high school graduate, 3 = some college, 4 = associate’s degree, 5 = college graduate, 6 = graduate degree. The income measure is the answer to the following question: How hard is it for you to acquire US$ 100 for personal spending? On a scale from 1 to 5 (with 1 = very hard and 5 = not hard at all). ESS denotes the effective sample size for each parameter. It is an estimate of the number of independent samples from the posterior (Kruschke, 2015). The Gelman-Rubin statistic $\hat{R}$ offers a quantitative approach to assess whether chains converge to the same posterior (Gelman and Hill, 2007). By comparing the variability between chains with the variability within chains, $\hat{R}$ determines convergence. When $\hat{R}$ < 1.1, it indicates that the chains have converged.

**Table 8:** Bayesian regression: loss aversion.

		95% credible interval			
	Posterior mean	Lower	Upper	ESS	*R*
1 = Buddhism	0.0349	−6.1718	7.2170	35,000	1.1
(As opposed to relig.unaffiliated)					
1 = Catholicism	1.3297	−5.4555	26.7009	36,142	1.7
(As opposed to relig.unaffiliated)					
1 = Hinduism	0.2134	−4.9561	5.5676	29,339	1.1
(As opposed to relig.unaffiliated)					
1 = Islam	−0.1979	−10.4458	6.2164	30,055	1.2
(As opposed to relig.unaffiliated)					
1 = Judaism	−0.9791	−23.0155	4.7533	29,639	1.5
(As opposed to relig.unaffiliated)					
1 = Mormon	−0.2112	−6.9053	5.1903	30,274	1.1
(As opposed to relig.unaffiliated)					
1 = Protestantism	0.0242	−6.1599	5.4469	25,475	1.1
(As opposed to relig.unaffiliated)					
1 = female	−0.5599	−12.5067	5.1643	26,829	1.4
(As opposed to male)					
1 = Ethnicity other than white	0.1363	−5.2873	6.7732	27,180	1.1
1 = High school graduate	−0.0668	−7.5544	6.4801	32,281	1.1
(As opposed to GED holder)					
Mothers’ education	0.2724	−6.1519	8.1111	35,179	1.1
Fathers’ education	0.1700	−5.1460	6.0198	44,898	1.0
Income measure	0.0962	−5.7029	6.2967	35,055	1.1
1 = any siblings	−0.1383	−5.8296	5.6060	36,306	1.1
(As opposed to only child)					
Big five openness	0.0087	−5.6509	5.8972	29,482	1.1
Big five conscientiousness	−0.0731	−4.7409	4.7676	32,257	1.1
Big five extraversion	0.0871	−4.6731	5.7799	32,627	1.1
Big five agreeableness	−0.4466	−6.6941	3.7128	33,427	1.1
Big five neuroticism	−0.2462	−6.4964	5.2540	34,053	1.1
Constant	1.6689	−7.2484	10.4929	34,187	1.2
Observations	110				

For all religious affiliation dummy variables, the baseline category are the religiously unaffiliated. Mother’s and father’s education are measured on a scale from 0 to 6, with 0 = less than high school or unknown, 1 = some high school, 2 = high school graduate, 3 = some college, 4 = associate’s degree, 5 = college graduate, 6 = graduate degree. The income measure is the answer to the following question: How hard is it for you to acquire US$ 100 for personal spending? On a scale from 1 to 5 (with 1 = very hard and 5 = not hard at all). ESS denotes the effective sample size for each parameter. It is an estimate of the number of independent samples from the posterior (Kruschke, 2015). The Gelman-Rubin statistic $\hat{R}$ offers a quantitative approach to assess whether chains converge to the same posterior (Gelman and Hill, 2007). By comparing the variability between chains with the variability within chains, $\hat{R}$ determines convergence. When $\hat{R}$ < 1.1, it indicates that the chains have converged.

The estimated sample sizes (ESS) are above 20,000 for all independent variables and constants in all three regressions presented. They provide an estimate of the number of independent samples obtained from the posterior (Kruschke, 2015). While there are no formal rules or tests regarding the adequate number of ESS for stable estimates (Baldwin and Larson, 2017), larger sizes are needed for higher levels of precision (Jackman, 2009). The Gelman-Rubin statistic offers a quantitative approach to assess whether chains converge to the same posterior (Gelman and Hill, 2007). By comparing the variability between chains to the variability within chains, $\hat{R}$ can be used to assess convergence. A value of the statistic <1.1 is frequently used to claim that all chains in the model have converged. Despite the high number of chains (16) employed in the estimations, this criterion of $\hat{R}$ < 1.1 is not met for all regressors and constants.

A possible interpretation of Bayesian regression results focuses on Bayesian credible intervals. These indicate parameter values with the highest probability, given the data and priors. If the credible interval does not contain zero, this can be interpreted as a variable with a positive or negative effect on the dependent variable. However, this is not the case for any of the independent variables in any of the three regressions presented here. In addition, the 95% credible interval for effects are wide, suggesting substantial uncertainty around the estimates. This confirms the results from Bayesian and frequentist analysis of variance reported in this research, suggesting no differences in economic preference parameters between the religions analyzed here.

## 5 Discussion

This exploratory research note tested possible differences in economic preference parameters (i.e., time preference, risk attitude, and loss aversion) between followers of different religions. It used experimentally elicited, incentivized measures of preference parameters and also included information on subjects’ personality traits as control variables in regressions. Bayesian and classical ANOVA analysis results suggest no differences between preference parameters analyzed in this research for the different religions, and these results were confirmed in Bayesian regression analysis when also controlling for a host of background variables.

These findings confirm some results from the scant previous research using incentivized choice tasks, such as the research by Chai et al. (2010), who reported no differences between Muslim and Christian participants in risk attitude and time preference. Similarly, Benjamin et al. (2016) report no effects of religious priming on subjects’ time preference, although they found that priming made Catholic participants less risk-averse compared to the religiously unaffiliated. However, they contradict the findings by Ayifah et al. (2022), who found that any religious affiliation is negatively correlated with the probability of engaging in a risky bet in Ghana, as well as the findings by Kahsay et al. (2022) for Ethiopia, who used an instrumental variables approach to address the possible endogeneity of religious affiliation and found the opposite, i.e., an increase of risky choices in a lottery task. Prinz et al. (2014) analyzed the responses from a student sample for Big Five personality traits and their correlations with incentivized risk-taking behavior. They found no statistically significant correlations, a result the present study confirms. However, in contrast to this research’s findings, Engle-Warnick et al. (2020) report a positive correlation between their conscientiousness and risk aversion measures.

Due to the multitude of risk and time preference measures, such as hypothetical questions on willingness to take risks (Barsky et al., 1997; Renneboog and Spaenjers, 2011; Paglieri et al., 2013), self-assessments of risk attitudes (Miller, 2000; Bartke and Schwarze, 2008), actual asset holdings (Halek and Eisenhauer, 2001; Bucciol and Zarri, 2017; León and Pfeifer, 2017), or the performance of investors’ portfolio performance (Firth et al., 2023) used in the other studies presented in the literature review, comparisons to the results presented in this study are difficult. Similarly, the personality traits measures used in the extant summarized literature show a wide conceptual variation, ranging from personality traits scales to classifications based on chat contents analysis (Engle-Warnick et al., 2020). Methodologically, none of the studies uses Bayesian methods, which might also contribute to the differences in outcomes reported in this research. However, the fact that findings across different studies vary significantly suggests potential issues with the consistency and validity of these constructs or differing effects of the same religion in different countries, affected by aspects of culture other than religion. For the preference parameters, different constructs and measurement tools used in the existing literature may not capture the same underlying phenomena, leading to divergent results. Frederick et al. (2002) discussed time preference and discounting, and Dohmen et al. (2011) underscored the importance of measurement consistency in risk attitudes. Future research on the topic might result in convergence in results, thanks to advances in conceptual measures of preference parameters and the use of more sophisticated statistical analysis methods. For the control variables, no differences were found between the preferences of male and female subjects. This research confirms the findings of Nelson (2015), who found that reported gender differences are „small “when analyzing effect sizes, and Filippin and Crosetto (2016), who found that they are task-specific.

For all results presented here, readers should acknowledge the small sample size, dictated by the need to provide monetary incentives for participation. Recent research by Brañas-Garza et al. (2023) suggests that experimentally elicited time preference is largely identical, regardless of the provision of monetary incentives, which might enable researchers to work with appropriately powered sample sizes in the future. Secondly, as pointed out by Benjamin et al. (2016) and the literature they cite, religion is likely an endogenous variable in the sense that it is correlated with other (unobservable) factors that affect preferences. However, the priming techniques they propose as a solution recently have faced scrutiny of their own, although the debate remains open with respect to possible causes (see, among others, Sherman and Rivers, 2021 for a discussion). While instrumental variables for religious affiliation have been suggested as a solution to this endogeneity problem and have been employed in previous research (Kahsay et al., 2022), weak instruments can potentially result in unreliable coefficient estimates and inference (see, for example, Andrews et al., 2019). Finally, a major limitation of this study is that group sizes for some religions are very small. Future research could, for example, be carried out in other countries where most individuals follow one of the religions that are underrepresented in this data set.

## Data availability statement

The raw data supporting the conclusions of this article will be made available by the authors, without undue reservation.

## Ethics statement

The studies involving humans were approved by Human Subjects Committee of the Faculty of Economics, Business Administration and Information Technology at the University of Zurich. The studies were conducted in accordance with the local legislation and institutional requirements. The participants provided their written informed consent to participate in this study.

## Author contributions

DB: Conceptualization, Data curation, Funding acquisition, Investigation, Methodology, Project administration, Resources, Software, Validation, Writing – original draft, Writing – review & editing.

## Footnotes

^1^Hoffmann (2013) provides an overview of the effects of religion on individual behavior in experiments with strategic interaction (i.e., games), such as public good games. Benjamin et al. (2016) provide an overview for experiments focusing on group identity effects of religion. Finally, there is also a strand of literature in corporate finance that uses regional variation in religious affiliation or the concentration of religious sites in an area around firms’ headquarters to find effects of religion on risk-taking behavior, such as Hilary and Hui (2009), Kumar (2009), Schneider and Spalt (2016), Li and Xu (2020), and Cebula and Rossi (2021). Since they are not relevant for the research question analyzed here, they are not summarized in more detail in the literature survey.
