# Supplementary material for: Religion, personality, or none of them? Exploratory evidence on their correlations with economic preference parameters
Source: Front Psychol. 2024 Aug 7;15:1361910. doi: 10.3389/fpsyg.2024.1361910 (PMC11338016; doi:10.3389/fpsyg.2024.1361910)
Supplement: Supplementary file 1 [file Data_Sheet_1.docx]

**Appendix A: Additional results**

**Table A1. Correlation matrix**

|  | Buddh. | Cath. | Hinduism | Islam | Judaism | Morm. | Protest. | Female | Non-white | High school | Mat.ed. | Pat.ed. | Income | Sibl. | Openness | Conscientiousness | Extraversion | Agreeableness | Neuroticism |
| --- | --- | --- | --- | --- | --- | --- | --- | --- | --- | --- | --- | --- | --- | --- | --- | --- | --- | --- | --- |
| Buddhism | 1 |  |  |  |  |  |  |  |  |  |  |  |  |  |  |  |  |  |  |
| Catholicism | 0.0303 | 1 |  |  |  |  |  |  |  |  |  |  |  |  |  |  |  |  |  |
| Hinduism | -0.025 | -0.0533 | 1 |  |  |  |  |  |  |  |  |  |  |  |  |  |  |  |  |
| Islam | -0.0506 | -0.1081 | -0.0186 | 1 |  |  |  |  |  |  |  |  |  |  |  |  |  |  |  |
| Judaism | 0.185 | 0.0382 | -0.016 | -0.0325 | 1 |  |  |  |  |  |  |  |  |  |  |  |  |  |  |
| Mormonism | -0.025 | -0.0533 | -0.0092 | -0.0186 | -0.016 | 1 |  |  |  |  |  |  |  |  |  |  |  |  |  |
| Protestantism | -0.1191 | -0.2542 | -0.0438 | -0.0888 | -0.0765 | -0.0438 | 1 |  |  |  |  |  |  |  |  |  |  |  |  |
| Female | -0.0838 | 0.0611 | 0.1069 | 0.119 | 0.0745 | -0.0858 | -0.0224 | 1 |  |  |  |  |  |  |  |  |  |  |  |
| Non-white | -0.0515 | -0.0733 | 0.0907 | -0.2052 | -0.1768 | -0.1012 | -0.049 | -0.0306 | 1 |  |  |  |  |  |  |  |  |  |  |
| High school | -0.0122 | 0.0198 | 0.0366 | 0.0742 | 0.0639 | -0.2508 | -0.042 | 0.0679 | 0.0755 | 1 |  |  |  |  |  |  |  |  |  |
| Mother's education | 0.0747 | 0.1472 | 0.1026 | 0.0141 | 0.1236 | -0.1364 | -0.0508 | -0.008 | -0.1505 | 0.1084 | 1 |  |  |  |  |  |  |  |  |
| Father's education | 0.0971 | 0.1691 | 0.1429 | -0.0236 | 0.1211 | -0.122 | -0.1941 | 0.1651 | -0.1787 | 0.0967 | 0.4207 | 1 |  |  |  |  |  |  |  |
| Income measure | 0.0069 | -0.0025 | -0.1619 | 0.0836 | -0.0068 | -0.1619 | -0.0923 | 0.2566 | -0.0601 | 0.0862 | -0.0473 | -0.0321 | 1 |  |  |  |  |  |  |
| Any siblings | 0.0084 | 0.0603 | 0.041 | -0.0513 | 0.0716 | -0.224 | 0.1288 | 0.029 | 0.1997 | 0.2895 | -0.0059 | -0.1277 | 0.0648 | 1 |  |  |  |  |  |
| Openness | -0.1194 | -0.1223 | 0.0695 | 0.0202 | -0.0018 | 0.096 | 0.1589 | 0.1048 | 0.1327 | -0.0511 | -0.0063 | -0.0883 | -0.0781 | 0.0649 | 1 |  |  |  |  |
| Conscientiousness | -0.0516 | 0.0485 | -0.1082 | -0.1561 | 0.0657 | -0.0457 | 0.1898 | 0.0435 | 0.1967 | 0.0044 | 0.1072 | -0.0573 | 0.0777 | 0.0975 | 0.3801 | 1 |  |  |  |
| Extraversion | -0.015 | 0.0645 | 0.116 | 0.0957 | -0.0596 | 0.0159 | 0.0194 | 0.0773 | 0.0281 | -0.1489 | 0.1526 | -0.0892 | 0.061 | -0.0054 | 0.3226 | 0.3264 | 1 |  |  |
| Agreeableness | -0.0584 | 0.0424 | -0.0517 | -0.1182 | -0.0133 | -0.0517 | 0.112 | 0.2082 | 0.2043 | 0.0781 | 0.1106 | -0.0014 | 0.1791 | 0.1335 | 0.3466 | 0.5457 | 0.2784 | 1 |  |
| Neuroticism | 0.0227 | -0.0446 | 0.1078 | 0.1856 | 0.0617 | 0.0861 | 0.0062 | -0.0633 | -0.1723 | -0.1634 | -0.0858 | 0.142 | 0.0395 | -0.1613 | -0.1791 | -0.3176 | -0.234 | -0.3301 | 1 |

| **Table A2. Parental education** | | | | |
| --- | --- | --- | --- | --- |
| **Mother's education:** | | **Father's education:** | |  |
| Less than high school/unknown | 18.18% | Less than high school/unknown | 24.55% |  |
| Some high school | 7.27% | Some high school | 7.27% |  |
| High school | 21.82% | High school | 19.09% |  |
| Some college | 18.18% | Some college | 11.82% |  |
| Associate's degree | 7.27% | Associate's degree | 6.36% |  |
| Bachelor's degree | 12.73% | Bachelor's degree | 15.45% |  |
| Graduate degree | 14.55% | Graduate degree | 15.45% |  |
|  | | | | |

**Table A.3 Classical analysis of variance**

The following table presents the results of classical (frequentist) ANOVA for risk attitude, time preference, and loss aversion.

| **ANOVA – risk attitude** | | | | | | | | | | | |
| --- | --- | --- | --- | --- | --- | --- | --- | --- | --- | --- | --- |
| **Cases** | | **Sum of Squares** | | **df** | | **Mean Square** | | **F** | | **p** | |
| Religions |  | 51.194 |  | 5 |  | 10.239 |  | 1.765 |  | 0.127 |  |
| Residuals |  | 591.769 |  | 102 |  | 5.802 |  |  |  |  |  |
|  | | | | | | | | | | | |
| *Note.*  Type III Sum of Squares | | | | | | | | | | | |
| **ANOVA – time preference** | | | | | | | | | | | |
| **Cases** | | **Sum of Squares** | | **df** | | **Mean Square** | | **F** | | **p** | |
| Religions |  | 107.245 |  | 5 |  | 21.449 |  | 0.674 |  | 0.644 |  |
| Residuals |  | 3247.163 |  | 102 |  | 31.835 |  |  |  |  |  |
|  | | | | | | | | | | | |
| *Note.*  Type III Sum of Squares | | | | | | | | | | | |
| **ANOVA – loss aversion** | | | | | | | | | | | |
| **Cases** | | **Sum of Squares** | | **df** | | **Mean Square** | | **F** | | **p** | |
| religion |  | 10.511 |  | 5 |  | 2.102 |  | 0.589 |  | 0.708 |  |
| Residuals |  | 364.035 |  | 102 |  | 3.569 |  |  |  |  |  |
|  | | | | | | | | | | | |
| *Note.*  Type III Sum of Squares | | | | | | | | | | | |

In all cases, the null hypothesis that there is no difference between group means cannot be rejected at conventional significance levels, suggesting no differences in preference parameters between the religions.

**Table A.4 Kruskal-Wallis tests**

The following table presents the results of Kruskal-Wallis tests of the hypothesis that several samples are from the same population for risk attitude, time preference, and loss aversion. Since this test is a multi-sample generalization of the two-sample Wilcoxon (Mann-Whitney) rank-sum test, it is an option to test for differences in means if the data are ordinal (i.e. if one is not willing to make the argument that parametric tests are reasonably robust to violations of the normality assumption, such as outlined in Norman (2010) or Knief and Forstmeier (2021)).

In all cases, the null hypothesis cannot be rejected at conventional significance levels, suggesting no differences in preference parameters between the religions.

| **Variable** | **Without ties** | **With ties** |
| --- | --- | --- |
| Risk attitude | Χ^2^(7) = 10.508 probability = 0.1616 | Χ^2^(7) = 10.877 probability = 0.1441 |
| Time preference | Χ^2^(7) = 4.890 probability = 0.6734 | Χ^2^(7) = 4.926 probability = 0.6690 |
| Loss aversion | Χ^2^(7) = 4.444 probability = 0.7274 | Χ^2^(7) = 4.603 probability = 0.7083 |

**Appendix B: Experimental instructions and personality scale**

All data utilized in this article were gathered through an online experiment involving undergraduate students from both two- and four-year colleges in California. The subjects were recruited through online channels and on-campus efforts. The online experiment was conducted using the eQuestionnaire platform. During the experiment, participants first provided their informed consent, then followed instructions for choice questions, received information on payment procedures, and were briefed on how decisions affecting payments would be determined. For risk attitude questions, one row was randomly selected for payment for each participant, while for time preference questions, every tenth participant was chosen for payment, with subjects only being informed post-experiment if they were selected. In intertemporal choice questions, payment was determined by randomly selecting one row from the corresponding choice sheet, and only the selected subjects received payment. Participants were notified that payments from risk and intertemporal choice questions, including a show-up fee of US$ 10 would be sent immediately after the experimental session via certified mail and in the form of a check. Future payments would be sent three months later through certified mail in the form of a check.

Experimental instructions

Choice 1 (Eliciting risk attitude)

In the first choice situation, you decide between a safe payment and a coin toss. If the coin comes up tails, you will receive USD 10. If the coin comes up heads, you will receive nothing.

In the table below, there are several decisions between the coin toss and the safe payment. For the coin toss, you will receive USD 10 every time when it comes up tails. For the safe payment, the amount you will receive varies between the different decisions.

Please decide for every line if you prefer the coin toss or the safe payment. At the end of this experiment, one line will be randomly selected for your payment. Depending on your decision in that line, you will receive either the safe payment or a coin will be tossed and you will receive either USD 10 or nothing, depending if it comes up heads or tails.

Examples:

1. Assume that you choose the coin toss for lines 1 to 8 and the safe payment for lines 9 and 10. This means that you prefer a safe payment of USD 9 or USD 10 to a coin toss where you can gain USD 10 if the coin comes up tails, but for a safe payment of USD 8 or less, you prefer the coin toss.

2. Assume that you choose the coin toss for lines 1 to 2 and the safe payment for lines 3 to 10. This means that for a safe payment of USD 3 or more you forego the coin toss where you can gain USD 10 if the coin comes up tails.

Please make a decision for each of the following lines.

|  | Coin toss, heads = $0, tails: $10 | Safe payment of $X |
| --- | --- | --- |
| 1. | □ I choose the coin toss | □ I choose the safe payment of $1 |
| 2. | □ I choose the coin toss | □ I choose the safe payment of $2 |
| 3. | □ I choose the coin toss | □ I choose the safe payment of $3 |
| 4. | □ I choose the coin toss | □ I choose the safe payment of $4 |
| 5. | □ I choose the coin toss | □ I choose the safe payment of $5 |
| 6. | □ I choose the coin toss | □ I choose the safe payment of $6 |
| 7. | □ I choose the coin toss | □ I choose the safe payment of $7 |
| 8. | □ I choose the coin toss | □ I choose the safe payment of $8 |
| 9. | □ I choose the coin toss | □ I choose the safe payment of $9 |
| 10. | □ I choose the coin toss | □ I choose the safe payment of $10 |

Choice 2 (Eliciting time preference)

In this choice situation, you have to decide between an earlier and a later payment (Option A and Option B). If you choose Option A, you will receive a certain amount by registered mail now. If you choose Option B, you will receive USD 100 by registered mail in 3 months from now. The amount you can receive if you choose Option A varies between the different decisions.

For this decision situation, there is a 10 percent probability that you will be selected for payment.

In the following table, there are several decisions between the two options listed. At the end of this experiment, one line will be randomly selected for your payment. If you are selected to be paid, you will receive your payment either now or in three months from now, depending on your choice in the line selected for your payment, by registered mail.

Please make a decision for each of the following lines.

|  | Option A | Option B |
| --- | --- | --- |
| 1. | □ $5 today | □ $100 in 3 months |
| 2. | □ $10 today | □ $100 in 3 months |
| 3. | □ $15 today | □ $100 in 3 months |
| 4. | □ $20 today | □ $100 in 3 months |
| 5. | □ $25 today | □ $100 in 3 months |
| 6. | □ $30 today | □ $100 in 3 months |
| 7. | □ $35 today | □ $100 in 3 months |
| 8. | □ $40 today | □ $100 in 3 months |
| 9. | □ $45 today | □ $100 in 3 months |
| 10. | □ $50 today | □ $100 in 3 months |
| 11. | □ $55 today | □ $100 in 3 months |
| 12. | □ $60 today | □ $100 in 3 months |
| 13. | □ $65 today | □ $100 in 3 months |
| 14. | □ $70 today | □ $100 in 3 months |
| 15. | □ $75 today | □ $100 in 3 months |
| 16. | □ $80 today | □ $100 in 3 months |
| 17. | □ $85 today | □ $100 in 3 months |
| 18. | □ $90 today | □ $100 in 3 months |
| 19. | □ $95 today | □ $100 in 3 months |
| 20. | □ $100 today | □ $100 in 3 months |

Choice 3: Eliciting loss aversion

In the following task, you have to decide if you want to choose a coin toss or not. If you choose the coin toss, you can win or lose money. If you choose the coin toss and lose money, you have to use the thank-you payment of US$ 10 to cover your losses. If you do not choose the coin toss, nothing happens: you neither win nor lose money.

If you choose the coin toss, we will throw a coin at the end of today’s experiment, and your wins or losses will be as follows:

• If the coin comes up heads, you will win US$6

• If the coin comes up tails, you will lose US$X

The amount of money that you will lose if the coin comes up tails differs between the different rows. In the table below, please decide for each row if you choose the coin toss or not. Again, if you choose the coin toss and the coin comes up heads, you will win 6,000 won, but if the coin comes up tails, the corresponding amount you lose will be deducted from your gains at the end of the experiment.

At the end of the experiment, one of your choices will be randomly selected as the decision-that-counts for your payments, by rolling a die. As all choices are equally likely to be selected as the decision-that-counts, please think carefully if you choose the coin toss or not. According to your choice in the decision that will be selected as the decision-that-counts, we will either toss a coin that will determine your gains or losses, or nothing will happen.

|  | I **decline** the coin toss (nothing will happen) | I **choose** the coin toss |
| --- | --- | --- |
| Tails: You lose US$2 Heads: You win US$6 | □ | □ |
| Tails: You lose US$3 Heads: You win US$6 | □ | □ |
| Tails: You lose US$4 Heads: You win US$6 | □ | □ |
| Tails: You lose US$5 Heads: You win US$6 | □ | □ |
| Tails: You lose US$6 Heads: You win US$6 | □ | □ |
| Tails: You lose US$7 Heads: You win US$6 | □ | □ |

Personality traits measurement: Big Five 15-item short scale

In the next section, we list several characteristics that an individual might have. Most likely, you will completely agree that some characteristics describe you well, while others don't, and you will be undecided on still others. Again, please indicate the degree to which the following statements are true for you, on a scale from 1 (not like me at all) to 7 (very much like me).

Please indicate the degree to which the following statements are true for you, on a scale from 1 (not like me at all) to 7 (very much like me).

I am somebody who…

- Does a thorough job
- Is talkative
- Is sometimes rude to others
- Is original, comes up with new ideas
- Worries a lot
- Has a forgiving nature
- Tends to be lazy
- Is outgoing, sociable
- Values aesthetic, artistic experiences
- Gets nervous easily
- Does things efficiently
- Is reserved
- Is considerate and kind to almost everyone
- Has an active imagination
- Is relaxed, handles stress well
